#  

**DOI:** 10.1111/jcmm.17076

**Published:** 2022-01-08

**FIGURE 1:**
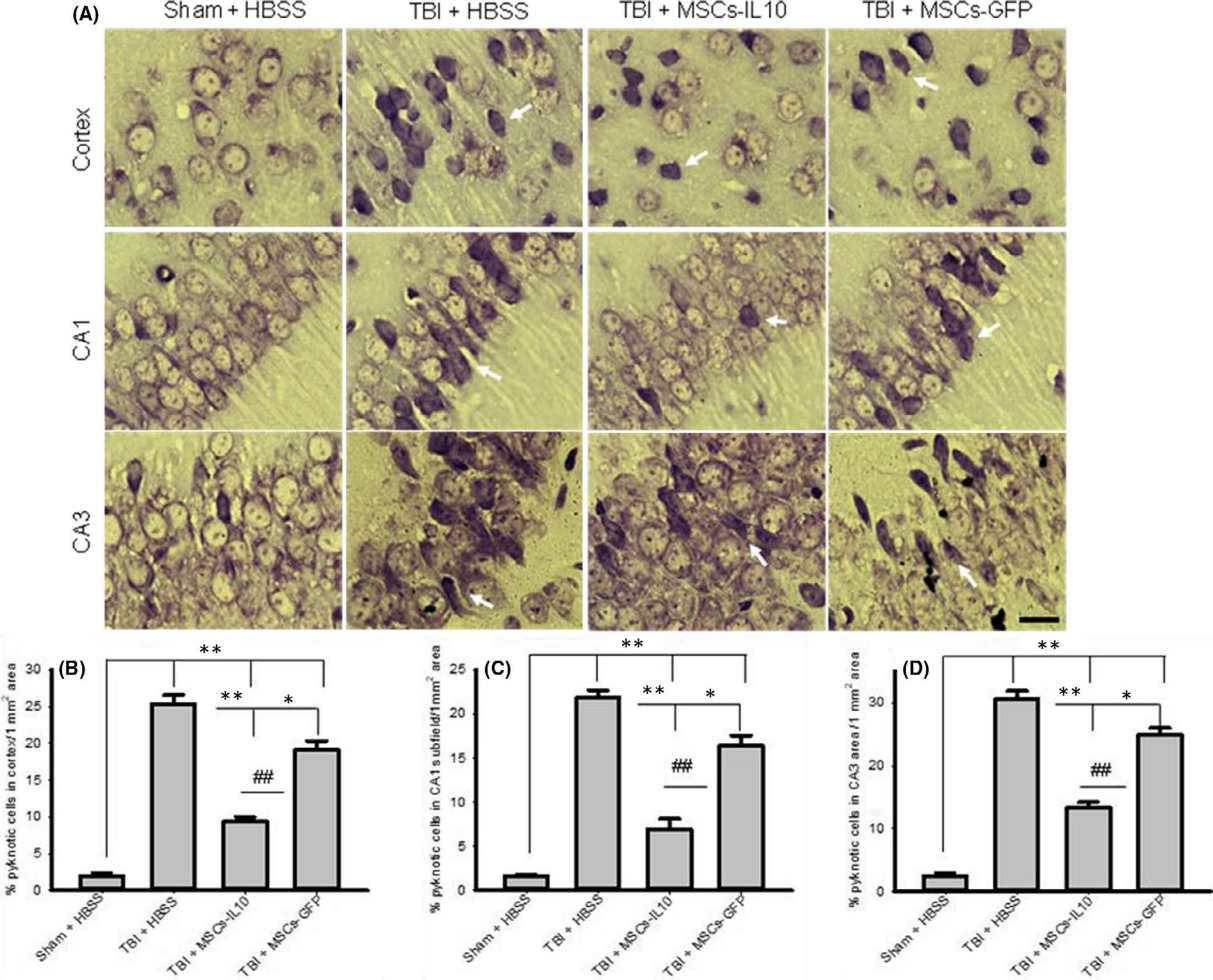
Transplantation of MSCs‐IL‐10 improved neuronal morphology greater than MSCs alone in the cortex and hippocampus of TBI rats. Rat brains were sectioned and stained with 0.1% Cresyl violet, and images were taken by compound light microscope (Olympus) with 100× objectives (total mag 1000×). (A) Representative photomicrograph of TBI rats showed increase in number of pyknotic or tangle‐like cells in the cortex, in the CA1 and CA3 subfields of hippocampus. (B‐D) Number of pyknotic cells were significantly decreased by transplantation of MSCs‐IL‐10 in comparison with TBI rats (*p* < 0.01) and with TBI + MSCs (*p* < 0.01). The greater reduction in pyknotic cells was observed in the case of MSCs‐IL‐10 rats. Arrows indicate pyknotic or tangle‐like cells. Scale bar indicates 100 µm and is applicable to other images. **p < 0.01 in comparison with TBI + HBSS, TBI + MSCs‐IL‐10 and TBI + MSCs; *p < 0.05 in comparison with TBI + MSCs; ^##^ *p* < 0.01 in comparison with TBI + MSCs

In Panchanan Maiti et al^1^, the panel of CA1‐TBI+MSCs‐IL‐10 is duplicated in Figure 1A. The correct figure is shown below. The authors confirm all results and conclusions of this article remain unchanged.
